# Survivin safeguards chromosome numbers and protects from aneuploidy independently from p53

**DOI:** 10.1186/1476-4598-13-107

**Published:** 2014-05-09

**Authors:** Ralf Wiedemuth, Barbara Klink, Katrin Töpfer, Evelin Schröck, Gabriele Schackert, Masaaki Tatsuka, Achim Temme

**Affiliations:** 1 Department of Neurosurgery, Section Experimental Neurosurgery/Tumor Immunology, University Hospital Carl Gustav Carus, TU Dresden, Fetscherstr. 74, 01307 Dresden, Germany; 2 Institute for Clinical Genetics, Medical Faculty Carl Gustav Carus, TU Dresden, Fetscherstr. 74, 01307 Dresden, Germany; 3 Department of Life Sciences, Faculty of Life and Environmental Sciences, Prefectural University of Hiroshima, Shoubara, Hiroshima, Japan

**Keywords:** Survivin, p53, p21^waf/cip^, ATM, DNA-PK_CS_

## Abstract

**Background:**

Survivin, a member of the inhibitor of apoptosis (IAP) gene family, has a dual role in mitosis and in apoptosis. It is abundantly expressed in every human tumor, compared with normal tissues. During mitosis Survivin assembles with the chromosomal passenger complex and regulates chromosomal segregation. Here, we aim to explore whether interference with the mitotic function of Survivin is linked to p53-mediated G_1_ cell cycle arrest and affects chromosomal stability.

**Methods:**

In this study, we used HCT116, SBC-2, and U87-MG and generated corresponding isogenic p53-deficient cells. Retroviral vectors were used to stably knockdown Survivin. The resulting phenotype, in particular the mechanisms of cell cycle arrest and of initiation of aneuploidy, were investigated by Western Blot analysis, confocal laser scan microscopy, proliferation assays, spectral karyotyping and RNAi.

**Results:**

In all cell lines Survivin-RNAi did not induce instant apoptosis but caused polyplodization irrespective of p53 status. Strikingly, polyploidization after knockdown of Survivin resulted in merotelic kinetochore spindle assemblies, γH2AX-foci, and DNA damage response (DDR), which was accompanied by a transient p53-mediated G_1_-arrest. That p53 wild type cells specifically arrest due to DNA damage was shown by simultaneous inhibition of ATM and DNA-PK, which abolished induction of p21^waf/cip^. Cytogenetic analysis revealed chromosomal aberrations indicative for DNA double strand break repair by the mechanism of non-homologous end joining (NHEJ), only in Survivin-depleted cells.

**Conclusion:**

Our findings suggest that Survivin plays an essential role in proper amphitelic kinetochore-spindle assembly and that constraining Survivin’s mitotic function results in polyploidy and aneuploidy which cannot be controlled by p53. Therefore, Survivin critically safeguards chromosomal stability independently from p53.

## Background

Among the family of inhibitor of apoptosis proteins (IAPs), Survivin has received vast attention because it is highly expressed in cancer tissues and cancer cell lines [1,2]. The gene for Survivin, *birc5*, is located in 17q25, and gives rise to a dominant expressed isoform with a molecular weight of 16.5 kDa [1]. Compared to other IAPs involved in apoptosis inhibition, Survivin is devoid of C-terminal RING finger and contains only one baculoviral IAP repeat (BIR) domain [1,3]. First attempts to elucidate the molecular function of Survivin demonstrated an anti-apoptotic function mediated by its BIR domain [4-7]. Although Survivin originally has been described to inhibit apoptosis by blocking activated caspases [7,8] and smac/Diablo [9], it is now unanimously recognized that the main molecular function of Survivin is linked to the control of the spindle assembly checkpoint (SAC) and cytokinesis [10]. The mitotic localization of Survivin is consistent with proteins described as chromosomal passenger proteins (CPP) [11-13]. During early mitosis, Survivin associates around centromeres together with its CPP partners Aurora B, Borealin and inner centromere protein (INCENP) to build a chromosomal passenger complex (CPC) [12,14]. At this location the CPC is responsible for sensing and correcting non-bipolar microtubule-kinetochore interaction [15-17]. It is hypothesized that the CPC can resolve the problem of syntelic- or merotelic-attached chromosomes by freeing the microtubule-kinetochore assembly, which eventually leads to the activation of the spindle assembly checkpoint (SAC) and therefore prevents premature onset of anaphase [18-20]. When anaphase starts Survivin leaves the kinetochores but in telophase re-aggregates with its CPC partners at the polar end of microtubules demarcating the cleavage furrow [21,22]. At this location, the Aurora B kinase within the CPC phosphorylates proteins regulating the contractile actin-myosin ring such as MGCRacGAP [23].

Cells with impaired function of Survivin or of one of its CPP partners due to RNAi-mediated inhibition or expression of dominant-negative mutants showed comparable phenotypes (i.e., disturbed segregation of chromosomes and defective cytokinesis) [5,21,24-26].

Many reports focused on the role of Survivin’s IAP function in the control of p53-governed cell cycle checkpoints and induction of apoptosis. Early studies using HCT116 cells and HCT116^p21−/−^ cells revealed that interfering mitosis with a SurvivinC84A mutant led to polyploidy and increased cell death in both cell lines [5]. However, the isogenic cells with knockout of the cell cycle regulator p21^waf/cip^ showed an even greater fraction of polyploid cells containing more nuclei per cell. From the observation that p21^waf/cip^ colocalized with Survivin and activated caspase 3 in centrioles of HCT116 wild type cells transfected with a Survivin C84A mutant, and experiments showing p21^waf/cip^ cleavage after Survivin antisense-treatment, it was inferred that this CDK inhibitor might be regulated by the IAP-function of Survivin. In addition, the inactivation of Survivin by antisense or dominant-negative SurvivinT34A resulted in increased mdm2 cleavage in MCF-7 cells leading to accumulation of p53 and cell death [27]. Another study observed an increase in p21^waf/cip^ protein levels after interference with Survivin function which was accompanied by activation of p53 [28]. Additionally, Survivin knockout in mouse thymocytes triggered a p53-mediated growth arrest but resulted in a p53-independent cell death [29].

So far it has been shown by various methods that the loss of Survivin function severely affects cell division. Yet, it has remained largely unclear why loss of Survivin in some cell lines leads to activation of p53, subsequent p21^waf/cip^ mediated cell cycle arrest and eventually causes cell death.

In this study, we decipher the direct effects but also a subordinate phenotype after Survivin RNAi in three different cell lines and their p53-deficient isogenic counterparts. Our results support published conclusions about Survivin’s function as a chromosomal passenger protein and uncovers the induction of an ATM and DNA-PK_CS_ mediated DNA damage response (DDR) after knockdown of Survivin in p53-deficient cells and wild type cells. Interestingly, cells with knockdown of Survivin developed polyploidy accompanied by the appearance of merotelic kinetochore-microtubuli assemblies in mitosis eventually resulting in DNA double strand breaks. Yet, cells managed to repair damaged chromosomes by non-homologous end joining recombination (NHEJ) resulting in aberrant chromosomes.

The appearance of merotelic kinetochore-microtubuli assemblies, chromosomal breaks and, the induction of DDR as well as the activation of p53 and induction of p21^waf/cip^ in cells with knockdown of Survivin displaying high grade polyploidy conclusively proves that an induction of a p53-dependent G_1_-arrest is a consequence of heavily disturbed mitosis. In conclusion, our results highlight the important role of Survivin as a chromosomal passenger protein which in concert with its molecular partners Aurora B, INCENP and Borealin, protects cells independently from p53 function from polyploidy and chromosomal instability.

## Results

### Knockdown of Survivin leads to a p53-independent development of polyploid cells

**Figure 1:**
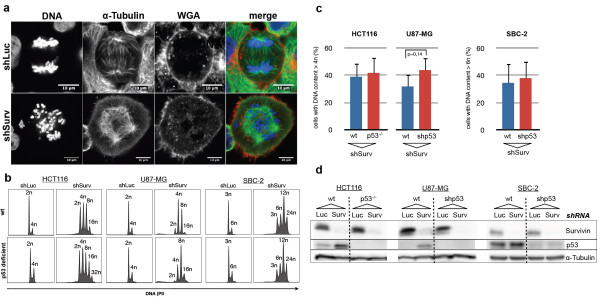
**Knockdown of Survivin leads to polyploidy in p53-positive tumor cells and isogenic cell lines deficient for p53.** HCT116, SBC-2, U87-MG and isogenic HCT116^p53−/−^, SBC-2^shp53^, and U87-MG^shp53^ cells were transduced with shRNAs targeting Survivin (shSurv) or Luciferase (shLuc). **a:** Representative indirect-immunofluorescence images of transduced SBC-2 cells. The shLuc-transduced cell shows a normal bipolar spindle formation in anaphase. The shSurv-transduced cell shows multipolar spindles and increased numbers of chromosomes. Microtubules (green), membranes (red) and chromosomes (blue). Magnification bars: 10 μm. **b:** FACS-analyses of cells with knockdown of Survivin depicting the appearance of polyploid cells in wild type and p53-deficient cells. **c:** Quantitative analysis of the polyploidy phenotype (i.e. cells with DNA contents >4n or >6n for SBC-2) of HCT116, U87-MG and SBC-2 and corresponding p53-negative cells. Data represents mean values and standard error of the mean (SEM). **d:** Western blot analysis of whole cell lysates demonstrating knockdown of Survivin and accumulation of p53 in Survivin-depleted HCT116, U87-MG and SBC-2 wild type cells but not in controls (shLuc) or p53-deficient cells.

In order to corroborate the relationship between Survivin and p53 and its downstream effector p21^waf/cip^ we analyzed the impact of Survivin knockdown on a panel of three cell lines with loss of p53 function and their isogenic p53-positive counterparts. We used HCT116^p53−/−^ colorectal carcinoma cells, and also newly generated SBC-2^shp53^ and U87-MG^shp53^ cells with a shRNA-mediated stable knockdown of p53. As controls served HCT116 wild type cells and isogenic U87-MG, SBC-2 cells transduced with a shRNA targeting firefly luciferase. Western blot analyses revealed an efficient knockdown of p53 in U87-MG and SBC-2 cells and demonstrated the accumulation of p53 in shLuc cells following treatment with the DNA-damaging drug doxorubicin (see Additional file 1). After knockdown of Survivin we detected in all six cell lines significant polyploidization and multipolar spindles in mitosis whereas all cell lines transduced with the control shRNA remained unaffected (Figure 1a, b). Interestingly, there were no major differences in the amount of cells displaying genomes >4n after Survivin-RNAi when comparing p53 wild type and p53-deficient cells in U87-MG and HCT116 cells and higher than 6n in SBC-2 cells (triploid cell line) (Figure 1c). Yet, we constantly observed that the grade of polyploidy was higher in p53-deficient cell lines when compared to p53-positive controls indicating a faster cell cycle progression rate (Figure 1b). When we tested the expression of p53 in Western blot analysis it became obvious that wild type cells accumulated p53 after Survivin-RNAi whereas HCT116^p53−/−^, U87-MG^shp53^, and SBC-2^shp53^, as expected, exhibited no accumulation of p53 (Figure 1d). These results indicate that the polyploidy following Survivin-RNAi is independent from p53 function, although Survivin-RNAi affects the steady state levels of p53 in wild type cell lines.

### The gradual increase in polyploidy in tumor cells with knockdown of Survivin is accompanied by activation of p53 and increased p21^waf/cip^ expression levels

Since it was of special interest whether stable loss of Survivin protein is compatible with life we performed clonogenic survival assays. Notably, all cell lines with stable knockdown of Survivin showed after a cultivation period of 3 weeks a dramatic decrease in clonogenic survival irrespective of p53 status when compared to shLuc-transduced cell lines (see Additional file 2a). Interestingly, after knockdown of Survivin no activation of effector caspase 3 was observed in p53-positive HCT116 and U87-MG as well as in p53-negative HCT116^shp53^ and U87-MG^shp53^ cells, respectively (see Additional file 2b, c).

However, when we assessed gross fragmentation of DNA it became obvious, that cell populations with knockdown of Survivin contained increased fractions of cells with hypodiploid DNA content when compared to shLuc controls (see Additional file 2d). Yet further AnnexinV/propidium iodide stainings at time points 24 h, 48 h after RNAi showed no significant difference in the fraction of apoptotic cells irrespective of p53 status when shSurv cells were compared to shLuc-transduced controls. Only at 72 h after Survivin-RNAi we observed an increase in the fraction of dead cells and a negligible increase in the fraction of apoptotic cells when compared to the shLuc controls which again was independent from p53 status (see Additional file 2e, f). In conclusion in our experimental setting knockdown of Survivin did not result in an apoptotic phenotype.

**Figure 2:**
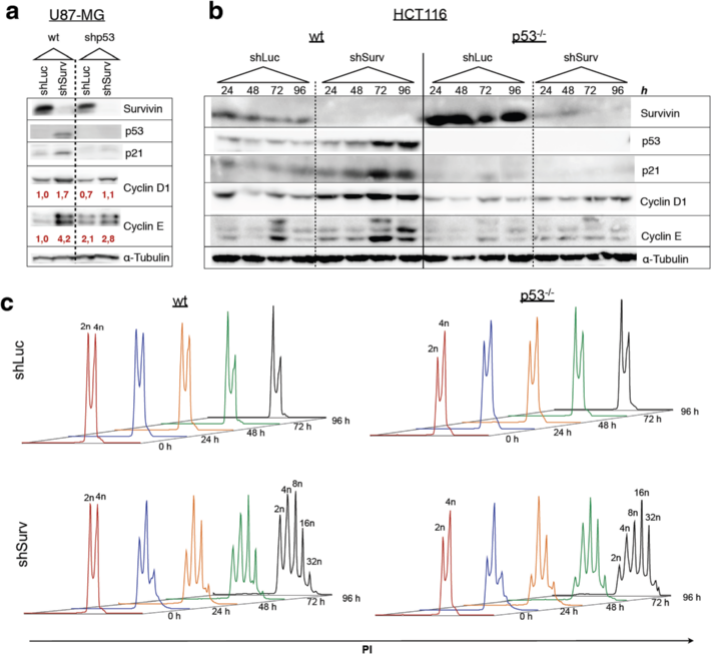
**Accumulating DNA contents after knockdown of Survivin leads to transient cell cycle arrest in p53-positive tumor cells. a:** Western blot analysis of U87-MG glioma cells and U87-MG^shp53^ 72 h after transduction of a shRNA targeting Survivin demonstrating an increase in steady state protein levels of p53, p21^waf/cip^. The increase in Cyclin D1 and Cyclin E protein levels in p53-positive wild type cells indicate a cell cycle arrest. A p21^waf/cip^–mediated cell cycle arrest is not observed in shLuc-transduced control cells or in isogenic U87-MG^shp53^ cells with knockdown of Survivin. Red values depict densitometric estimation of protein levels, normalized to Tubulin. **b:** Western blot analysis and parallel DNA-analysis of HCT116 cells at different time points after RNAi showing a gradual increase in p53, p21^waf/cip^, Cyclin D1 and Cyclin E protein levels only in p53-positive cells (wt) after knockdown of Survivin but not in corresponding HCT116^p53−/−^ cells and shLuc controls. **c:** DNA analyses of HCT116 and HCT116^p53−/−^ after transduction showing time-dependent gradual increase in polyploidy in Survivin-depleted cells. The amount of DNA content (n) in the cell fractions is indicated.

When we analyzed total cell lysates of U87-MG cells it became apparent that the knockdown of Survivin, besides the increase of p53 steady state expression, caused an induction of p21^waf/cip^ (Figure 2a), whereas U87-MG^shp53^ cells were not affected. In addition, a concomitant increase in the protein expression levels of Cyclin D1 and Cyclin E indicated a G_1_ cell cycle arrest in p53-positive U87-MG cells following Survivin-RNAi (Figure 2a). Interestingly, we also observed a slight increase in Cyclin D1 and Cyclin E protein expression levels after knockdown of Survivin in U87-MG^shp53^ cells, suggesting an attenuated cell cycle progression independent from p53 function. In order to assess whether an activation of the p53/p21^waf/cip^ axis occurs as an immediate effect or develops together with a gradual increase in polyploidy, we analyzed in parallel the cell cycle and expression of cell cycle proteins of HCT116 wild type and HCT116^p53−/−^-cells at different time points after RNAi (Figure 2b, c). In line with previously published data showing inhibitory effects of p53 on Survivin transcription [30,31] we found decreased Survivin protein steady state levels in shLuc-transduced HCT116 wild type cells when compared to shLuc-transduced HCT116^p53−/−^ cells at all investigated time points (Figure 2b). Yet, in the aforementioned U87-MG cells we did not find decreased expression levels of Survivin in U87-MG cells when compared to U87-MG^shp53−/−^ cells which is likely caused by mdm2-augmented degradation of p53 in those PTEN-negative cell lines [32].

The knockdown of Survivin in HCT116^p53−/−^-cells revealed, as expected, no signs of p53 expression or induction of p21^waf/cip^, but an increase in the grade of polyploidy over time. Furthermore, it only weakly increased Cyclin D1 expression and did not alter the expression levels of Cyclin E when compared to the shLuc-transduced controls. In HCT116 wild type cells the knockdown of Survivin clearly demonstrated a time-dependent increase in the accumulation of p53 protein as well as a gradual increase in expression levels of p21^waf/cip^ (Figure 2b). Furthermore, progressively increased Cyclin D and E protein levels indicate a G_1_ arrest. In conclusion these results show a time-dependent increase of markers of G_1_ cell cycle arrest in p53-positive cells after knockdown of Survivin which was paradoxically accompanied by an ongoing endoreplication of the genome leading to the development of HCT116 cells bearing genomic DNA contents in the range of 4n to 32n (Figure 2c). Yet, the question arises why p53-positive cells with knockdown of Survivin display molecular signs of a G_1_ cell cycle arrest, but are still capable of replicating their genomes.

### Polyploidy in p53-positive cells correlates with p21^waf/cip^-mediated cell cycle arrest and attenuates S-phase entry

**Figure 3:**
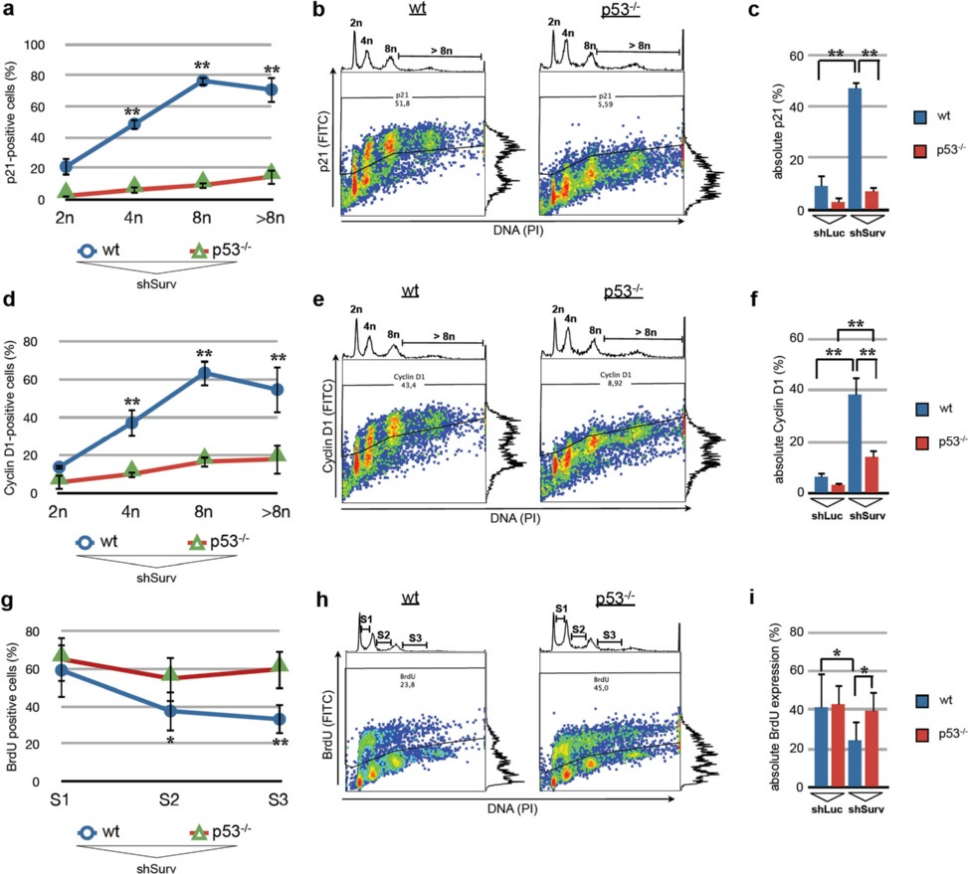
**Increasing DNA contents after Survivin-RNAi are linked to a p53-dependent transient cell cycle arrest and to an attenuated entry into S-phase. a-c:** Combined PI-staining of DNA and p21^waf/cip^ in HCT116 and HCT116^p53−/−^ cells 72 h after transduction of shRNA-vectors. **a:** Graph showing the mean values and SEM of p21^waf/cip^-positive cell fractions after knockdown of Survivin at different DNA contents ranging from 2n to greater than 8n (**p < 0.01). **b:** Representative FACS analysis showing p21^waf/cip^ staining in HCT116 (wt) and HCT116^p53−/−^ cells. **c:** Quantitative analysis of the induction of p21^waf/cip^ in total HCT116 and HCT116^p53−/−^ cell populations after transduction of shSurv and shLuc control, respectively (**p < 0.01; n = 4) **d-f:** Combined PI-staining of DNA and Cyclin D1 in HCT116 and HCT116^p53−/−^ cells after transduction of shRNA-vectors. **d:** Graph showing the mean values and SEM of Cyclin D1-positive cell fractions after knockdown of Survivin at different DNA contents (**p < 0.01). **e:** Representative FACS dot blots showing Cyclin D1 analyses in HCT116 (wt) and HCT116^p53−/−^ (p53^−/−^) cells **f:** Quantitative analysis of the induction of Cyclin D1 expression in total HCT116 and HCT116^p53−/−^ cell populations after transduction of shSurv and shLuc control, respectively (**p < 0.01). **g-i:** BrdU-incorporation analysis. **g:** Graph showing the mean values and SEM of BrdU-positive cell fractions after knockdown of Survivin in S-phases of cells (S1 to S3). Note, that despite p21^waf/cip^ and Cyclin D1 accumulations HCT116 cells with DNA content >4n are still able to incorporate BrdU. **h:** Representative FACS dot blots showing BrdU stainings after Survivin knockdown. **i:** Effect of Survivin knockdown on BrdU incorporation in total HCT116 and HCT116^p53−/−^cell populations and the corresponding shLuc controls. Data represents mean values and SEM (*p < 0.05; **p < 0.01).

In order to address the above mentioned question and to correlate the grade of polyploidy (4n and higher) of the cells directly to regulators of the cell cycle and DNA-synthesis we employed a simultaneous BrdU-incorporation/DNA-staining protocol as well as concomitant DNA/p21^waf/cip^, and DNA/Cyclin D1 staining protocols for flow cytometry. Interestingly, an increase in p21^waf/cip^ (Figure 3a, b) as well as Cyclin D1-positive cell fractions (Figure 3d, e) after knockdown of Survivin showed a significant and direct correlation with increasing polyploidy in p53-positive HCT116 cells when compared to isogenic p53^−/−^-cells (p < 0.01), indicating that cells containing higher DNA content had a greater probability of undergoing G_1_ cell cycle arrest.

When analyzing total cell populations after Survivin-RNAi it became apparent that the p53-positive cells contain significant increased fractions of p21^waf/cip^ (Figure 3c) and Cyclin D1-positive cells (Figure 3f) when compared to p53-positive cells transduced with shLuc control vector (each p < 0.01) and p53^−/−^-cells after knockdown of Survivin, respectively (each p < 0.01). Interestingly, after knockdown of Survivin in p53^−/−^-cells we observed a considerable smaller and significant increase in the fraction of Cyclin D1 positive cells in the whole cell population when compared to p53^−/−^-shLuc controls (p < 0.01), yet without increase in p21^waf/cip^ expression suggesting a slight p53-independent effect on cell cycle progression (Figure 3f).

On the other hand, the BrdU-incorporation analysis revealed that substantial fractions of p53 wild type cells as well as p53^−/−^-cells with knockdown of Survivin were still able to enter S-phases of cells with DNA contents of 4n and higher (Figure 3g, h). In contrast to Cyclin D1 and p21^waf/cip^-expression levels we found a significant decrease in BrdU-incorporation levels in p53-positive cells when compared to HCT116^p53−/−^ (p < 0.05), which was inversely correlated to increase in polyploidy (Figure 3g).

When we compared the BrdU incorporation levels of the total cell populations we revealed a significant decrease in the BrdU-positive cell fraction of p53-positive cells with knockdown of Survivin compared to shLuc controls (p < 0.05) and even to p53^−/−^-cells with knockdown of Survivin (p < 0.05) (Figure 3i).

In conclusion, these results demonstrate an association of Survivin-RNAi-induced polyploidy with p53 activation and induction of p21^waf/cip^. However, p53 activation and induction of p21^waf/cip^ after knockdown of Survivin did not hinder entry into S-phase, which indicates a transient cell cycle arrest. Yet, the molecular cause of this observed transient cell cycle arrest remains obscure.

### Survivin-RNAi leads to an increase in merotelic spindle attachments, DNA breaks, induction of a DNA damage response, and chromosomal instability

**Figure 4:**
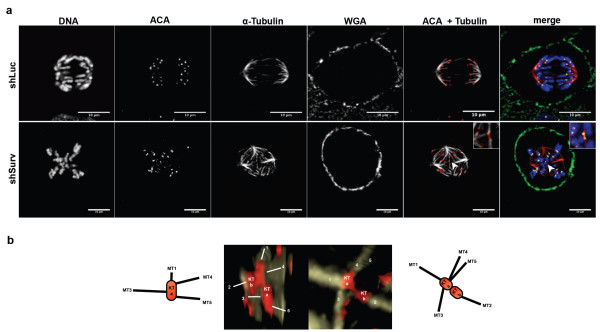
**Survivin controls kinetochore-microtubuli attachments. a:** Indirect-immunofluorescence images of mitotic SBC-2 wild type cells, transduced with shLuc or shSurv, respectively. The shLuc-transduced cell shows a normal bipolar spindle assembly during anaphase. The depicted shSurv-SBC-2 cell is dodecaploid and displays abnormal spindle formation in metaphase with a poly-merotelic connected chromosome (arrowhead; inset). **b:** Different angles of a 3D-reconstructed z-stack of the same merotelic attachment shown in **a:** demonstrating binding of up to four microtubli from three different directions to a single kinetochore (KTa). MT, microtubuli (red); KT, kinetochore; (yellow); membrane (green).

A recent study demonstrated an increase in merotelic attachments in multipolar cells [33]. Staining of SBC-2 wild type cells after knockdown of Survivin with anti-tubulin and anti-centromere-antibodies (ACA) revealed multiple spindles with spindle fibers bound to diverse aligned metaphase chromosomes, thereby causing merotelic and moreover poly-merotelic (arising from multiple spindle poles) connections to chromosomes (Figure 4, and see Additional file 3). In particular, in cells with higher polyploidy kinetochores of sister chromatids bound to spindles emanating from several spindle poles were constantly observed (see dodecaploid cell in Figure 4).

**Figure 5:**
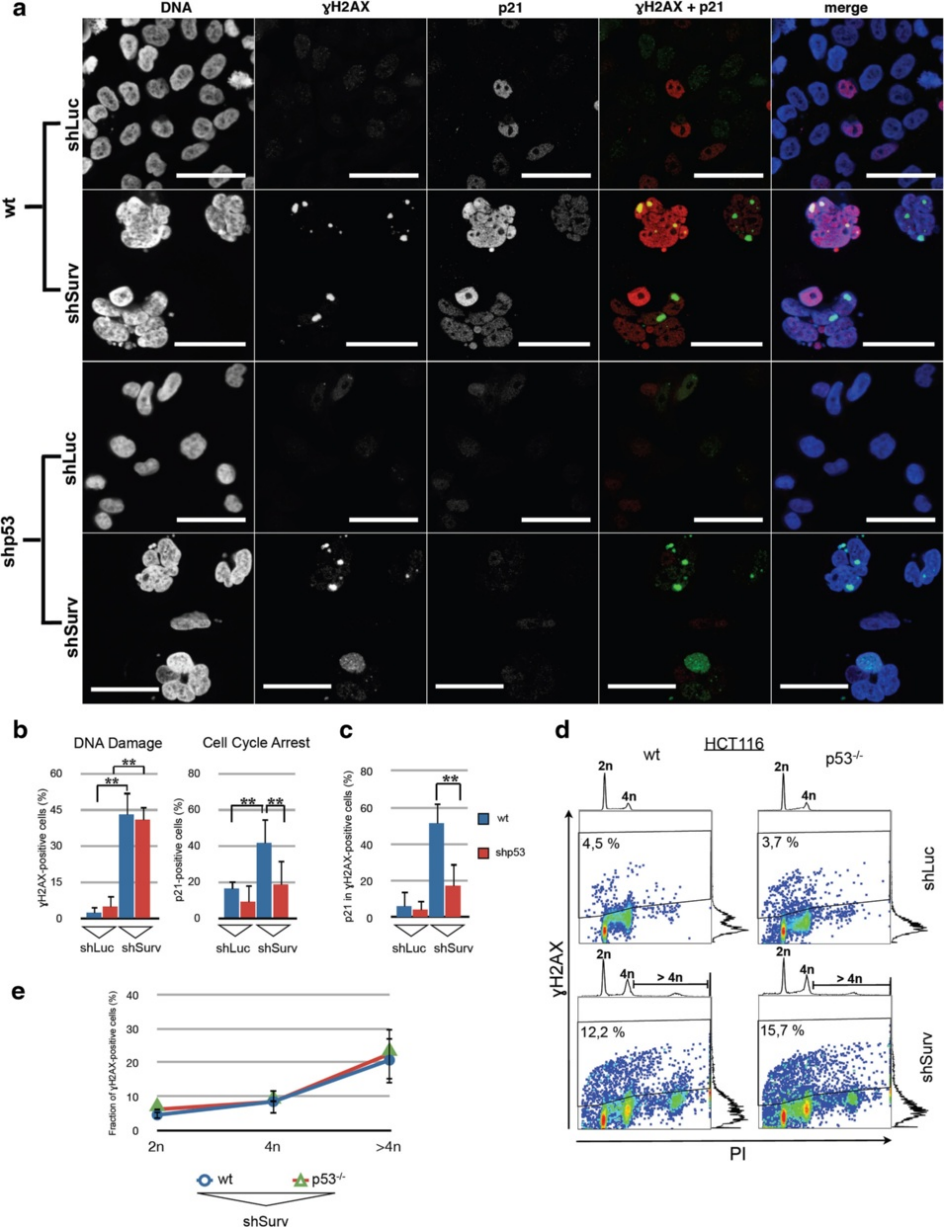
**Knockdown of Survivin results in DNA damage and p53-dependent induction of the cdk-inhibitor p21**^**waf/cip**^**. a-c:** Indirect-immunofluorescence analysis of SBC-2 and SBC-2^shp53^ cells, transduced with shLuc and shSurv, respectively. **a:** Representative images of simultaneous p21^waf/cip^ and ɣH2AX stainings showing ɣH2AX-foci only in polyploid nuclei of shSurv-SBC-2 cells. Magnification bar 50 μm. **b:** Quantification of ɣH2AX and p21^waf/cip^ expression. Depicted are the mean values and SEM (n = 3; **p < 0.01). Knockdown of Survivin causes DNA-damage irrespective of p53 status whereas induction of p21^waf/cip^ is p53-dependent. **c:** Quantification of ɣH2AX positive cells with simultaneous p21^waf/cip^ expression. Note the increase in p21^waf/cip^ expression linked to DNA damage in SBC-2 cells with functional p53 (p < 0.01). **d:** Representative FACS-analysis showing an increase in ɣH2AX staining in Survivin-depleted HCT116 cells compared to shLuc control, irrespective of p53-status. **e:** Distribution of ɣH2AX expression in wild type and HCT116p53^−/−^ cells in dependence of DNA content. The increase in ɣH2AX-positive cell fractions is independent from p53 status and directly correlates to the gradual increase in polyploidy.

Since non-resolved merotelic attachments can cause chromosomal breaks and subsequently a DNA damage response [34], we hypothesized that the observed high abundance of merotelic attachments due to the knockdown of Survivin might be linked to the activation of p53 and its downstream effector p21^waf/cip^. In order to detect probable resulting chromosomal breaks on the molecular level we analyzed the expression of γH2AX, a marker for DNA-double strand breaks (DSB) in SBC-2 p53-positive cells and SBC-2^shp53^-cells after Survivin-RNAi. By using indirect immunofluorescence analysis and quantification of γH2AX positive cells it became obvious, that a significant fraction of cells had definitely acquired DNA-damages after knockdown of Survivin irrespective of p53 status (mean ± SEM, wt/shLuc: 2.4% ± 2.7%; wt/shSurv: 43.0% ± 9,5%, p < 0.01; shp53/shLuc: 4,8% ± 5,1%; shp53/shSurv: 40,8% ± 5.9%, p < 0.01) (Figure 5a, b). The observed γH2AX foci were 1 to 3 μm in diameter and appeared only in multinucleated cells marking a moderate number of DNA lesions per cell (1 to 5 DSB/cell) (Figure 5a). As expected, a significant increased nuclear expression of p21^waf/cip^ after Survivin-RNAi, indicative of a cell cycle arrest, was only observed in p53 wild type cells when compared to SBC-2^shp53^ cells (p < 0.01)(Figure 5b). Furthermore, quantitative analysis of SBC-2 p53 wild type cells revealed that about 50% of γH2AX-positive cells (mean ± SEM, wt/shSurv: 51.4% ± 11.5%) showed induction of p21^waf/cip^, as detected in the nuclei of cells. On the other hand SBC-2^shp53^ cells (mean ± SEM, shp53/shSurv: 17.2% ± 12.3%) showed significantly less (p < 0.01) p21^waf/cip^ signals in γH2AX-positive nuclei (Figure 5c).

In line with these results additional FACS-assisted analysis of γH2AX expression confirmed increased γH2AX expression preferentially in polyploid HCT116^p53−/−^ and corresponding isogenic p53-positive cells after knockdown of Survivin whereas transduction of the shLuc control had no effect on the levels of γH2AX-positive cells (Figure 5d). DNA damage gradually increased together with the DNA content of cells with knockdown of Survivin (Figure 5e).

**Figure 6:**
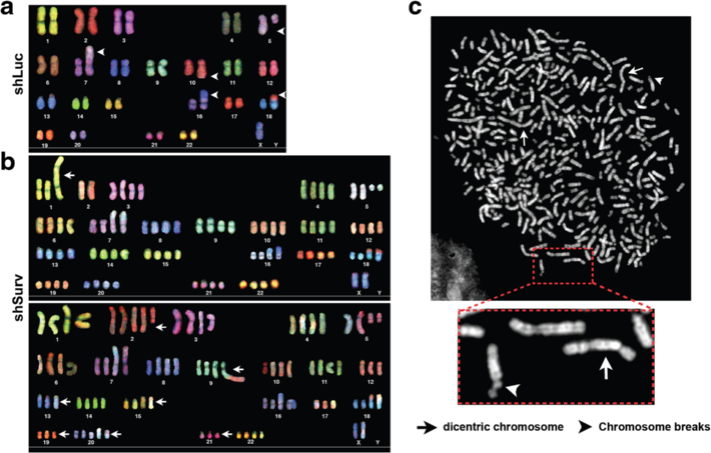
**Survivin protects cells from chromosomal instability. a:** SKY-Analysis reveals chromosomal instability (gain of numerical and structural chromosomal aberrations as well as polyploidy) in Survivin-depleted HCT116^p53−/−^ cells 72 h after retroviral transduction. Representative karyogram of shLuc-transduced HCT116^p53−/−^ cell with hypodiploid (2n-) karyotype 45,X,-Y,t(5;7)(q13;pter), der(10)dup(10)(q23.1q26.1)t(10;16)(q26.1;q23), der(16)t(8;16)(q13;pter), der(18) t(17;18)(q21.3;pter) (arrowheads). **b:** Exemplary karyograms of Survivin-depleted HCT116^p53−/−^ cells showing nearly tetraploid karyotypes (87,XX), harboring the aberrations found in shLuc, but also additional aberrations. Upper karyogram: additional dicentric chromosome 1 (arrow) and loss of two chromosomes 2. Lower karyogram: additional structural aberrations del(2)(q13), der(9)t(9;12), der(15)t(15;17), der(20)t(3;20) and loss of chromosome 13, 19, 21 (arrows). **c:** DAPI-stained metaphase of HCT116^p53−/−^ cells with knockdown of Survivin showing a hypohexadecaploid (16n-) karyotype (360 chromosomes) with dicentric chromosomes (arrows) and chromosome breaks (arrowheads).

In order to investigate whether the high number of observed merotelic and poly-merotelic chromosomal attachment to the spindle apparatus and the resulting DSBs eventually cause structural chromosomal aberrations, we analyzed HCT116^p53−/−^-cells using Giemsa and DAPI staining. Furthermore, we performed spectral karyotyping (SKY). HCT116^p53−/−^-cells transduced with the control vector (shLuc) displayed a stable hypodiploid karyotype (2n-, chromosome number 45, 44 autosomes and one X chromosome) in the SKY analyses with recurrent chromosomal aberrations described for the HCT116^p53−/−^ cell line in the NCI and NCBI’s SKY/M-FISH and CGH Database (2001), (http://www.ncbi.nlm.nih.gov/sky/skyweb.cgi) including deletion of the long arm of chromosome 5, unbalanced translocations between chromosomes 5 and 7, 8 and 16, as well as between 17 and 18, and a derivative chromosome 10 with duplication of material of the long arm of chromosome 10 and translocation of material from chromosome 16 (45,X,-Y,t(5;7)(q13;pter), der(10)dup(10)(q23.1q26.1)t(10;16) (q26.1;q23), der(16)t(8;16)(q13;pter), der(18)t(17;18)(q21.3;pter)) (Figure 6a). In contrast, in HCT116^p53−/−^ cells with knockdown of Survivin, the majority of metaphases analyzed showed a near tetra-, octa-, or even hexadecaploid karyotype (85–91, 151–172, and ~ 300 chromosomes per metaphase). Additionally to the chromosomal aberrations already present in HCT116^p53−/−^ shLuc, SKY-analysis marked chromosomal instability with additional structural chromosomal aberrations in all metaphases analyzed, such as translocations, loss of whole chromosomes, dicentric chromosomes, chromosome fragments, or truncated chromosomes (Figure 6b). Analysis of Giemsa- and DAPI-stained metaphases of HCT116^p53−/−^ shSurv cells revealed constantly dicentric and ring chromosomes, which can occur due to DSB repair (Figure 6c, and see Additional file 4). No dicentric and ring chromosomes were detected in shLuc-transduced cells. Moreover, we identified a higher frequency of chromosomal breaks in shSurv-HCT116^p53−/−^ cells compared to HCT116^p53−/−^ shLuc cells (mean breaks/cell for shLuc 0.25; mean breaks for shSurv 1, p < 0.05). So far our data suggest that knockdown of Survivin causes defective cytokinesis (leading to polyploidy), failures in the SAC, and increased DSBs (leading to chromosomal instability with structural chromosomal aberrations and aneuploidy).

### Survivin-RNAi induced DNA damage response is mediated through combined action of ATM and DNA-PK sensor kinases

**Figure 7:**
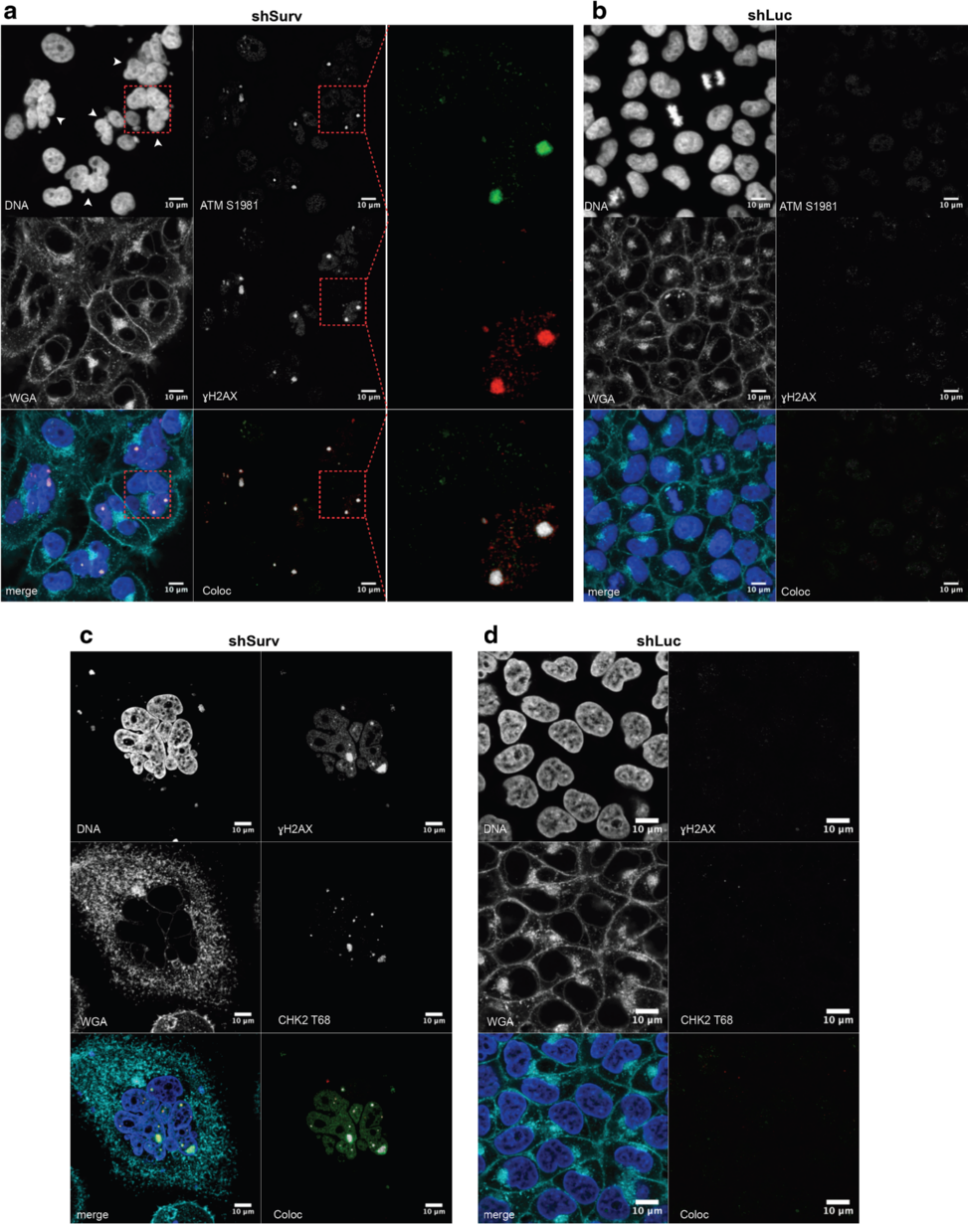
**Activation of ATM and CHK2 at DNA lesions in SBC-2 cells with knockdown of Survivin. a:** Representative immunofluorescence analysis of SBC-2 cells, with knockdown of Survivin and stained for activated ATM (ATM S1981) and ɣH2AX. Note double positive ɣH2AX, ATM S1981 loci indicative for DDR occurring in polyploid nuclei (arrowheads). Inlet showing magnification of indicated multinucleated cell with colocalized ɣH2AX and ATM S1981. **b:** Representative image depicting ɣH2AX, ATM S1981 staining in shLuc-transduced controls. Magnification bars: 10 μm. **c:** Representative image of SBC-2 cells after knockdown of Survivin showing the appearance of activated CHK2 (CHK2 T68) in ɣH2AX-positive DNA lesions. **d:** ɣH2AX and CHK2 T68 do not appear in shLuc-transduced SBC-2 cells. Magnification bars: 10 μm.

To analyze the connection between Survivin-RNAi and the induction of a DNA-damage response (DDR) we focused on *ataxia telangiectasia mutated* gene product (ATM) a sensor kinase involved in DSB repair. By using confocal laser scanning we detected activated ATM (ATM S1981) colocalized to γH2AX foci, with a diameter of 1–3 μm, in SBC-2 wild type cells with knockdown of Survivin (Figure 7a). Cells transduced with the shLuc control vector never displayed such γH2AX/ATM foci (Figure 7b). In line with this we detected similar γH2AX/ATM-foci in shSurv-U87-MG cells (see Additional file 5). Noteworthy, activated CHK2 phosphorylated at T68, a downstream target of ATM was colocalized with γH2AX in SBC-2 cells with knockdown of Survivin (Figure 7c) whereas no signals were found in shLuc control cells (Figure 7d).

**Figure 8:**
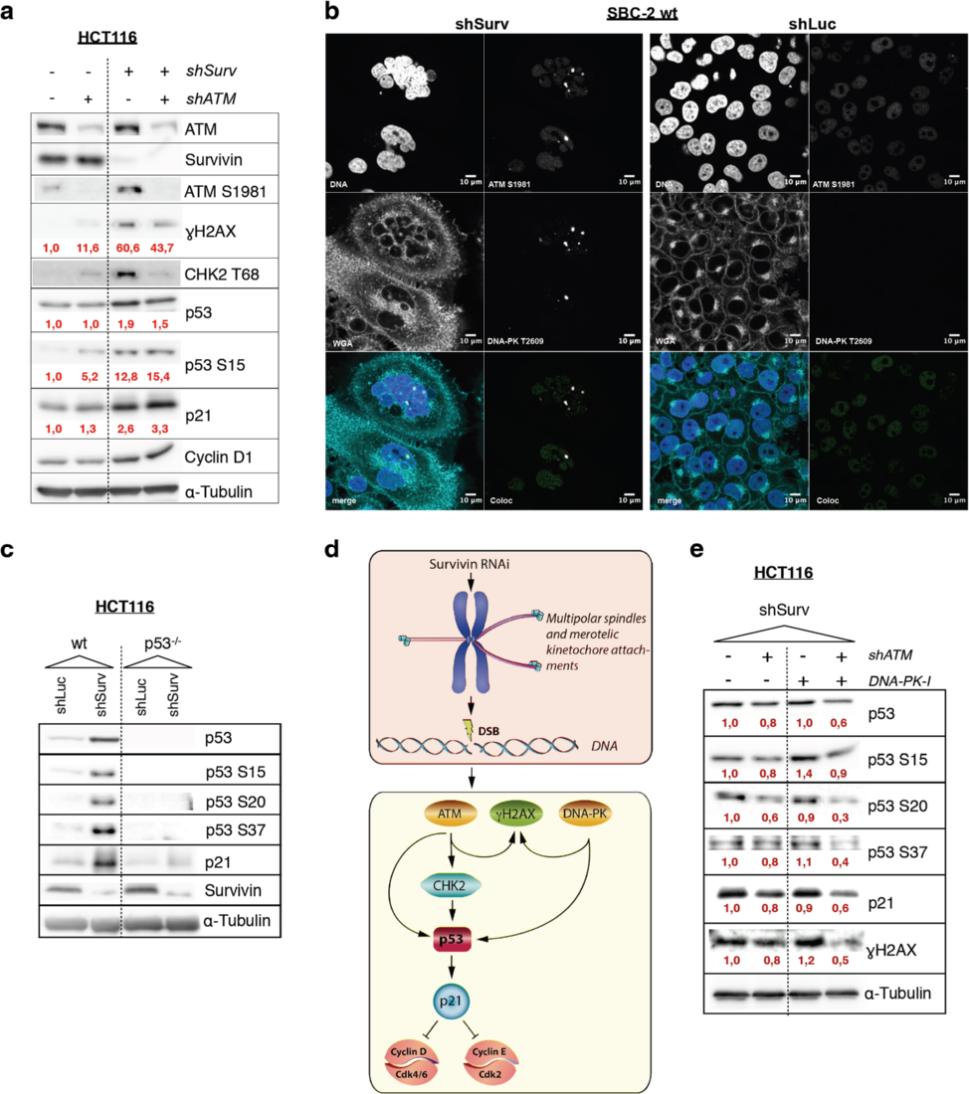
**ATM and DNA-PK**_**CS **_**induce cell cycle arrest after Survivin-RNAi. a:** Western blot analysis of HCT116 and HCT116^shATM^ total cell lysates following Survivin-RNAi (shSurv). Included are shLuc-transduced cells as control. Red values indicate densitometric estimation of protein levels, normalized to Tubulin. **b:** Indirect immunofluorescence analysis of transduced SBC-2 cells showing colocalisation of activated ATM (ATM S1981) and activated DNA-PK kinase (DNA-PK_CS_ T2609) in shSurv polyploid cells. **c:** Western blot of HCT116 and HCT116^p53−/−^ cell lysates showing analysis of p53 phosphorylation sites after Survivin knockdown. **d:** Proposed outline of Survivin RNAi phenotype and the observed DNA-damage response signaling. **e:** Analysis of HCT116 and HCT116^shATM^ cells transduced with shSurv and combined treatment with DNA-PK_CS_ inhibitor (DNA-PK-I, 10 μM Nu7026) or DMSO. Note combined inhibition of ATM and DNA-PK_cs_ results in a decreased accumulation of p53 and p21^waf/cip^ protein levels and reduction of γH2AX levels.

Our observations suggest that the activation of a transient p53/p21^waf/cip^ -dependent cell cycle arrest in G_1_ after Survivin knockdown occurs after mitotic defects and is linked to the CPP function of Survivin. To verify that the DDR and not the loss of Survivin’s IAP function accounts for p53 activation and subsequent induction of p21^waf/cip^ we generated HCT116 wild type cells with stable knockdown of ATM. We hypothesized that an inactivation of ATM should block phosphorylation of p53 by an activated ATM which in turn should abolish p21^waf/cip^ expression. Survivin-RNAi in HCT116 wild type cells caused, as expected, increased γH2AX protein expression levels accompanied by increased protein levels of p53, p53 phosphorylated at Serine 15 (p53^pSer15^), p21^waf/cip^ and Cyclin D1 when compared to shLuc control cells (Figure 8a). Furthermore, as anticipated, we detected activated ATM (ATM S1981) in these cells. Noteworthy, ATM S1981 most likely phosphorylates p53 at position S15 in HCT116 cells with knockdown of Survivin. Besides this, we also detected its downstream kinase CHK2 phosphorylated at T68 [35] (Figure 8a).

Remarkably, the inactivation of ATM in HCT116 cells (HCT116^shATM^) resulted after knockdown of Survivin in a strong decreased CHK2 phosphorylation at Thr68 and slightly decreased levels of γH2AX. On the other hand, it did not inhibit the activation of p53 and the subsequent induction of p21^waf/cip^ expression, respectively. Since our cytogenetic analysis revealed chromosomal translocations, we also investigated the role of DNA PK_CS_ DNA-sensor kinase, which plays a pivotal role in DNA repair by the mechanism of non-homologous end joining (NHEJ). Interestingly, we detected activated DNA-PK_CS_ colocalized with ATM S1981 positive foci in nuclei of cells following RNAi of Survivin (Figure 8b). In line with this Western blot analysis of p53 phosphorylation sites in shSurv-treated HCT116 cells revealed enhanced phosphorylation of p53 at S15, S20 and at position S37 indicative of activated ATM, CHK2 and DNA-PK_cs_ kinases [36,37] (Figure 8c). We therefore concluded that the DNA damage response in HCT116^shATM^ following Survivin-RNAi is also sensed and initiated by DNA-PK (see proposed sensor kinase signaling in Figure 8d). To conclusively prove that loss of Survivin CPP function (in controlling cytokinesis and SAC) is responsible for a transient cell cycle arrest we simultaneously blocked ATM (by stable RNAi) and DNA-PK_CS_ (using the specific inhibitor Nu7026) upstream of p53 and p21^waf/cip^. As depicted in Figure 8e only the combined inhibitions of both DNA damage sensor kinases led to a decrease in phosphorylation of p53 and subsequently to significantly decreased p21^waf/cip^ protein levels. In particular, simultaneous inhibition of DNA-PK_cs_ and ATM lead to a weaker decrease in S15 phosphorylation but strongly abrogated p53 phosphorylation at S20 and S37 and reduced protein levels of γH2AX. These results corroborate the involvement of DNA-PK_CS_ and ATM in the induction of the p53/p21^waf/cip^ mediated transient cell cycle arrest, DSB repair and NHJE in cells with knock down of Survivin.

## Discussion

### The RNAi-phenotype of Survivin confirms its role as chromosomal passenger protein and uncovers its essential role in DNA integrity

Precise chromosome segregation during mitosis requires the attachment of chromosomes to microtubules emanating from both poles of the spindle apparatus. Non-attachment of spindles to kinetochores is sensed by mitotic SAC proteins including CENP-E, Bub1, BubR1, Bub3, Mad1, Mad2 and Mps1. Although not all interactions of SAC proteins are fully understood, it is generally accepted that Mad2 represents the effector protein in charge that becomes activated at kinetochores. Mad2 inhibits the anaphase promoting complex/cyclosome (APC/C) by direct binding to Securin and Cyclin B, whose degradation is required for onset of anaphase [38,39]. Yet, during the stochastic attachment process of spindle-kinetochore binding, all but bipolar attachments need to be resolved to allow new rounds of attachments. Bipolarity of spindle kinetochore attachment is subjected to pulling forces of the mitotic spindle which is opposed by cohesion between the sister chromatids. Today it is generally suggested, that the CPC, especially its kinase Aurora B, cuts off microtubule binding due to lack of tension on the kinetochores [17,20]. This in turn leads to free kinetochores which activate the mitotic SAC [40] and allows a new round of kinetochore microtubuli attachments.

Our study confirmed that Survivin is essentially needed for cytokinesis but also for proper regulation of bipolar spindle attachment. Noteworthy, Survivin is not controlling cell cycle arrest as revealed by our spatial and temporal analysis of the developing Survivin-RNAi phenotype. We conclusively corroborate that the induction of a transient G_1_ arrest is a consequence of heavily disturbed mitosis, which is solely linked to Survivin’s role as CPP as discussed in detail below. Furthermore, cell death after knockdown of Survivin was not accompanied with increased levels of activated Caspase 3 or increase in the fraction of annexin V-positive cells but was linked to the gradual increase in polyploidy. This observation is consistent with other reports describing a mitotic catastrophe after interference with the function of Survivin challenging its proposed role as IAP during mitosis [41-43].

Our investigations reveal that the primary effect of the knockdown of Survivin, i.e. polyploidy and defective chromosomal segregation, develops independently from p53 and leads to DNA double strand breaks followed by DDR and enhanced chromosomal instability (Figure 8d). We conclude that the loss of Survivin at kinetochores in conjunction with multipolar spindles dramatically increases the likelihood of merotelic spindle kinetochore assemblies which cannot be resolved and finally leads to disrupted chromosomes. Yet, the DDR resulted in a transient p53/p21^waf/cip^ –mediated cell cycle arrest in G_1_ and cells managed to repair DSBs through NHEJ recombination. In line with this, the inhibition of another chromosomal passenger protein, Aurora-B, recapitulated the Survivin RNAi phenotypes (Wiedemuth et al., manuscript in preparation).

The fact that the transient p53/p21^waf/cip^-dependent cell cycle arrest was exclusively due to induction of a DNA damage response and not connected to the loss of IAP function of Survivin was proven by simultaneous inhibition of sensor kinases DNA-PK and ATM, which abolished activation of p53 and induction of p21^waf/cip^.

Our results have profound implications concerning a possible postmitotic G_1_ cell cycle arrest of tetraploid cells. Recent reports suggested a p53 dependent G_1_ checkpoint preventing tetraploid cells to enter S phase [44]. Until now, it has remained entirely unknown by which mechanism cells respond to tetraploidy in a p53-dependent manner. In particular, the results from our BrdU-incorporation assays suggest that tetraploidy occurring in cells with defective CPC but functional p53 does not prevent progression into the next S-phase. Although a significant fraction of cells suffer DSBs caused by poly-merotelic kinetochore assemblies, they managed to repair DSBs during a transient p53/p21^waf/cip^-mediated arrest and progressed in their cell cycle. If the same transient or eventually a stable cell cycle arrest occurs in tetraploid cells having an intact CPC or in cells with defects in the SAC remains to be investigated.

### The potential role of Survivin in radioresistance and tumor progression

Survivin has also been implicated in radiation resistance of tumor cells. After radiation Survivin accumulates in interphase nuclei of glioblastoma and colorectal carcinoma cells [45-47]. So far, the underlying mechanisms how Survivin protects cells from radiation is still not fully understood. It has been shown that interfering Survivin’s function by adenoviral overexpression of a dominant-negative mutant of Survivin (SurvivinT34A) leads to decreased DNA damage repair capacity in primary glioblastoma cells as detected by a DNA comet assay [45]. Furthermore, siRNA of Survivin leads to a decreased activity of DNA-PK_CS_ in LN229 glioma cells after radiation and to a concomitant increase in residual γH2AX foci per nucleus [46]. Strikingly, the same group mentioned in their supplementary data increased levels of phospho-ATM in non-irradiated SW480 colorectal carcinoma cells after Survivin-RNAi, which is in line with our results [47]. However, how the knockdown of Survivin affects repair of DSB after radiation still remains elusive since our SKY analysis shows that DNA repair, in particular NHEJ, does not require Survivin and does not even depend on p53.

So far the IAP function of Survivin in conjunction with increased expression levels in tumors has also been implicated to confer resistance to cellular stress and chemotherapy by inhibiting caspases [48], smac/DIABLO [9] and apoptosis inducing factor (AIF) [49]. In particular, ectopic overexpression of Survivin in the skin of transgenic mice has been described to confer resistance to UV-mediated apoptosis but also resulted in enhanced progression of skin tumors [50,51]. Various scientific articles emphasized a possible role of the IAP function of Survivin in tumor progression [52,53]. Yet, some studies demonstrated that overexpression of Survivin also results in a higher frequency of polyploid bone marrow and glioma cells [24,54]. Furthermore, a low frequency of polyploid cells was detected in normal human lung fibroblasts and CD34-positive hematopoetic stem cells with ectopic overexpression of Survivin [24]. Up to now, it is well known that the deregulated expressions or the altered stoichiometry of mitotic regulators, in particular Survivin’s partner Aurora B, increase the risk for carcinogenesis by induction of polyploidy and subsequent aneuploidy [55,56]. As a result of our study and in conjunction with recent reports describing that the overexpression of Survivin favors the progression from chemically induced papilloma to squamous cell carcinoma in K14-Survivin transgenic mice [57] and initiates hematological malignancies in GATA1-Sur transgenic mice after a single intraperitoneal injection of tumor promoting N-ethyl-nitrosourea [58], it appears also conceivable that besides Survivin’s IAP function deregulations in the mitotic function of Survivin favor the development of aneuploid cells. In the future it might be therefore worthwhile to investigate the impact of deregulated Survivin protein expression levels on genetic stability and carcinogenesis.

## Conclusions

In summary, we have investigated in depth the role of Survivin in control of chromosome segregation and chromosomal stability. Our data indicate that a transient p21^waf/cip^ –mediated cell cycle arrest after knockdown of Survivin is not connected to a proposed IAP function in mitosis, but instead represents a DDR phenotype linked to its role as CPP. Paradoxically our results indicate that p53 expression does not prevent aneuploidy when interfering with Survivin’s CPP function. Therefore, Survivin and its molecular partners in the CPC fundamentally control genetic stability of cells.

## Material and methods

### Cell culture and clonogenic survival

The hypodiploid colorectal carcinoma-derived HCT116 cells (kindly provided by B. Vogelstein, Johns Hopkins University, Baltimore) were cultured in RPMI medium (Life Technologies) supplemented with 10% FCS, 2 mM L-glutamine, 10 mM Hepes, 100 U/ml penicillin and 100 μg/ml streptomycin, whereas the near triploid cervix carcinoma cell line SBC-2 and the human embryonic kidney cells 293 T were maintained in Dulbecco’s modified Eagle medium containing 4.5 g/l glucose (Life Technologies) supplemented with 10% FCS, 10 mM HEPES, 100 U/ml penicillin and 100 μg/ml streptomycin. The diploid glioblastoma- derived U87-MG cell line and cultivation has been described before [59]. All cell lines were authenticated (Multiplexion GmbH, Heidelberg, Germany) and cultured at 37°C with 5% CO_2_. Clonogenic survival of transduced U87-MG, HCT116, SBC-2 cells and p53-deficient counterparts was tested by plating duplicates of 1000 cells/dish for every vector transduction onto 10 cm dishes. After 3 weeks, cells were stained with Giemsa and the number of clones was quantified. At least two independent experiments with similar results were performed for each cell line. For DNA-PK_cs_ inhibition cells were cultured, transduced and selected as described. 6 hours before cell lysis media containing 10 μM Nu7026 (Selleckchem) or DMSO were added to the cells.

### Retroviral shRNA vectors and transduction

For knockdown of target RNAs we used self-inactivating retroviral Moloney murine leukemia virus (MoMuLV) backbones pRVH-1-puro and pRVH-1-Hygro, both containing a H1 polymerase III promoter for the expression of shRNA molecules. All DNA oligonucleotides encoding shRNAs were synthesized with appropriate BamH I and Sal I restriction sites (Eurofins MWG Biotech) to allow ligation into pRVH-1 vectors. For knockdown of Survivin we used the recently described small hairpin RNA shSurvivin#433 [26] ligated into pRVH-1-puro. For the generation of U87-MG^shp53^, SBC-2^shp53^ and HCT116^shATM^ cells we used the shRNAs shp53 (upper strand: 5′-gatccccgactccagtggtaatctacttcaagagagtagattaccactggagtctttttggc − 3′, bottom strand: 5′-tcgagccaaaaagactccagtggtaatctactctcttgaagtagattaccactggagtcggg − 3′ [60]) and shATM (upper strand: 5′-gatccccgataccagatccttggagattcaagagatctccaaggatctggtatctttttggc − 3′, bottom strand: 5′-tcgagccaaaaagataccagatccttggagatctcttgaatctccaaggatctggtatcggg − 3′ [61]) ligated in pRVH1-Hygro. As negative control, a previously described RNA targeting sequence for luciferase mRNA (shLuc) was included during all experiments [59]. Transduction of retroviral shRNA vectors was performed as described previously [26]. For the generation of stable p53- (shp53) or ATM- (shATM) deficient cells, transduced cells were selected and cultured using 300 μg/ml Hygromycin B (Life Technologies).

### Indirect immunofluorescence analysis

Cells grown on glass slides were fixed for 20 min with 4% PFA. Then they were washed three times for 5 min in HBSS and stained with WGA-Texas Red (wheat germ agglutinin; Life Technologies) in HBSS (10 μg/ml) for 10 min followed by a permeabilization step with 5% BSA, 0.3% Triton-X-100 in PBS for 30 min. Cells were incubated with a primary antibody solution (5% BSA in PBS) for 2 h including monoclonal mouse anti-α-Tubulin (Sigma), monoclonal rabbit anti-p21^waf/cip^ (Cell Signaling), monoclonal rabbit anti-CHK2 T68 (Cell Signaling), monoclonal rabbit anti-ATM S1981 (Epitomics), monoclonal mouse anti-γH2AX (Millipore) and monoclonal mouse anti-DNA-PK_CS_ T2609 (Abcam), respectively. After three 5-min-washes with PBS, cells were subsequently incubated with a secondary antibody solution (sheep/goat anti-mouse Cy3-conjugated antibody, Dianova; sheep/goat anti-rabbit FITC-conjugated antibody, Dianova) for 1 hour followed by extensive wash cycles in HBSS. DNA counterstaining was accomplished using HBSS-Hoechst 33342 (0.1 μg/ml, Life Technologies) solution. Finally, cells were washed twice in HBSS and once in double distilled water, prior the examination by confocal laser scanning microscopy. Colocalization analyses (Coloc) were performed using Fiji’s “Colocalization” algorithm. The p21^waf/cip^ and ɣH2AX statistics were calculated after analyzing the average percentage of fluorescence-labeled cells in at least 3 random fields of shLuc-transduced cells and 6–17 random fields of shSurv-transduced cells, representing at least 570 cells per group. Statistical analysis was performed using Student’s t-test.

### Detection of merotelic kinetochore-microtubuli assemblies

Cells were rinsed with HBSS and stained with WGA-Texas Red in HBSS (10 μg/ml) for 10 min at 37°C followed by an incubation with Ca^2+^ buffer (100 mM PIPES pH 6.8; 1 mM MgCl_2_; 1 mM CaCl_2_; 0.5% Triton-X-100) for 7 min at 37°C and fixation with 4% PFA in PBS for 20 min at room temperature. After three 5-min-washes in PHEM buffer pH 6.9 (60 mM PIPES; 25 mM HEPES; 10 mM EGTA; 2 mM MgCl_2_), cells were permeabilized (5% BSA in PHEM; 0.3% Triton-X-100) for 30 min at room temperature, subsequently washed three times for 5 min in PHEM buffer and were then incubated at room temperature with the primary antibodies in blocking solution (5% BSA in PHEM buffer). Antibodies include mouse anti-α-Tubulin (1:1000; 1.5 h; Sigma) and human anti-centromeric antibody (ACA, 1:500, kindly provided by K. Conrad, TU Dresden, Germany). The primary antibodies were detected after three 5 min washes (PHEM buffer) with polyclonal sheep anti-mouse-Cy3 (1:100; 1 h; Dianova) and anti-human-FITC (undiluted; 30 min; AKLIDES ANA plus; Medipan) successively in blocking solution at room temperature in the dark. After three 5-min-washes in HBSS, DNA was counterstained with Hoechst 33342 (0.1 μg/ml).

Stained cells were imaged with a Leica SP5 inverse microscope (Leica, Wetzlar, Germany). Confocal images were collected at 405, 488, 543 and 594 nm with 63x NA1.4 or 40x NA1.25 objective lenses. Image acquisition, shutter, Z-axis position, laser lines, and confocal system were all controlled by Leica LAS AF software. Series in z-directions (Z-stacks) of single cells were obtained at 0.3 μm steps. Equivalent exposure conditions and scaling was used between controls and RNAi-depleted cells. Images were analyzed using Fiji software [62] (http://fiji.sc/Fiji). For analysis of merotelic attachments, the acquired images were processed in Fiji including background subtraction, contrast enhancement, smoothing and 3D reconstruction. A kinetochore was scored as being merotelic when it clearly connected two or more visible spindle fibers emanating from different poles in a z-stack or reconstructed 3D image.

### Western blot analysis

Cells were harvested and lysed in lysis buffer (10 mM Tris–HCl pH 8.0; 140 mM NaCl; 1% Triton-X-100). Equal amounts of proteins were loaded onto SDS polyacrylamide gels, separated by gel electrophoresis and blotted onto PVDF membranes. Subsequently, membranes were washed 3 times for 10 min in TBS plus 2% Triton X-100 and 0.5% Tween 20 (TBS-TT) and blocked with 5% milk powder or BSA. The membranes were probed with primary antibodies in blocking solution over night at 4°C including rabbit polyclonal anti-Survivin (R&D Systems), polyclonal mouse anti-p21^waf/cip^ (R&D Systems), monoclonal rabbit anti-p21^waf/cip^ (Cell Signaling), polyclonal goat anti-p53 (R&D Systems), monoclonal rabbit anti-p53 S15 (Abcam), polyclonal anti-p53 S20, polyclonal anti-p53 S37 (Cell Signaling), monoclonal mouse anti-α-Tubulin and monoclonal anti-Actin (Sigma), monoclonal rabbit anti-ATM S1981 (Epitomics), polyclonal rabbit anti-ATM (Merck), monoclonal rabbit anti-Caspase 3 (Cell Signaling), monoclonal rabbit anti-CHK2 T68 (Cell Signaling), polyclonal rabbit anti-Cyclin D1 (Santa Cruz), polyclonal rabbit anti-Cyclin E (Santa Cruz) and monoclonal mouse anti-γH2AX (Millipore). After incubation with the primary antibodies, the membranes were washed three times with TBS-TT and incubated for 1 hour at room temperature with appropriate secondary antibodies conjugated with HRP (Dako). The membranes were washed again and signal detection was carried out by chemiluminescence (Luminata Forte, Millipore) using a LAS3000 device. Western blots were quantified with Fiji’s “Gel Analyzer Tool” and densitometric values were normalized to Tubulin.

### Flow cytometry

Cell cycle and apoptosis was determined in a flow cytometer using PI-stained cells as described previously [26]. Analysis of raw data was performed using FlowJo software (Tristar, Inc.). For combined DNA-antibody staining 2 x 10^5^ cells/sample were permeabilized and blocked using 5% BSA in PBS plus 0.3% Triton X-100 for 30 min at room temperature. After centrifugation (1500 rpm, 4°C, 5 min) supernatant were discarded and cells incubated with monoclonal rabbit anti-p21^waf/cip^ (Cell Signaling), polyclonal rabbit anti-Cyclin D1 (Santa Cruz), monoclonal mouse anti-γH2AX (Millipore), polyclonal rabbit IgG (Santa Cruz) or monoclonal mouse IgG1 (Millipore) respectively, diluted in blocking solution for 2 h at room temperature. Primary antibodies were detected using FITC-conjugated goat anti-rabbit or FITC-conjugated sheep anti-mouse (Dianova) antibodies for 1 h at room temperature. Finally, DNA was counterstained with 20 μg/ml PI and 40 μg/ml RNase A in PBS for 30 min at room temperature in the dark.

For DNA-synthesis analysis cells were incubated with 10 μM BrdU (Sigma) and analyzed as described previously [26]. FACS data processing includes doublet discrimination and debris exclusion. All experiments were performed at least three times with similar results. Statistical analysis was performed with Student’s t-test. Annexin V staining has been performed according to manufacturer instruction (Life Technologies). Briefly, cells were washed once in PBS and trypsinized. After centrifugation (300 g, 8 min, 4°C) supernatant was removed and cells were washed again with PBS followed by re-centrifugation. Then, cell pellet (maximum 10^6^ cells) was resuspended in 100 μl of annexin V binding buffer and 5 μl of annexin V were added. After an incubation period of 15 min at room temperature samples were diluted with 400 μl of annexin V binding buffer and re-centrifuged. Finally, cell pellets were resuspended in 100 μl annexin V binding buffer. PI (1 μg/ml, Miltenyi) was automatically added right before FACS measurement using MACSQuant analyzer (Miltenyi).

### Cytogenetic analyses

HCT116^p53−/−^ shLuc and shSurv cells were analyzed using Spectral karyotyping (SKY), DAPI and Giemsa-staining. For preparation of metaphase chromosomes 72 h after transduction methotrexate (5 μg ml) was added for further 17 hours. Medium was removed and cells incubated in fresh medium with 150 μl BrdU-FUdR-solution at 37°C for 5 h. Cells were treated with colcemid for 60 min at a concentration of 0.035 μg/ml, incubated in 75 mM KCl for 20 minutes at 37°C, and fixed in a freshly prepared mixture of methanol/acetic acid (3:1) at room temperature. Cell suspension was dropped onto glass slides. Spectral karyotyping (SKY) analysis was performed as described previously [63]. SKY images of about at least 20 metaphase chromosomes per cell line stained with a mixture of 5 fluorochromes were captured using an DMRXA epifluorescence microscope (Leica GmbH, Wetzlar, Germany), HCX PL SAPO 63x/1.30 oil objective (Leica), SpectraCube® system (Applied Spectral Imaging, Migdal HaEmek, Israel), and SKYView® imaging software (Applied Spectral Imaging). Additionally, metaphase chromosomes were stained with DAPI and Giemsa and number of chromosomes (ploidy), chromosomal breaks, and chromosomal aberrations (ring and dicentric chromosomes) per metaphase were counted. Statistical analysis was performed with Student’s t test. Mann–Whitney-U test was carried out to compare frequencies of breaks as well as ring and dicentric chromosomes.

## Competing interests

The authors declare that they have no competing interests.

## Authors’ contributions

AT designed and supervised the study. RW, KT, BK and ES performed and analyzed most of the experiments. MT and GS analyzed and interpreted data. AT and RW wrote the manuscript with ideas and comments from the rest of the authors. All authors read and approved the final manuscript.

## Supplementary Material

Additional file 1 **This figure shows efficiency of p53 knockdown and response to doxorubicin treatment in U87-MG and SBC-2 cells.** Western blot analysis showing the knockdown of p53 in glioma and cervix cancer tumor cells. Note that U87-MG and SBC-2 cells accumulate p53 in response to DNA-damaging doxorubicin treatment (0.25 μg/ml) whereas no increase of p53 is observed in U87-MG^shp53^ and SBC-2^shp53^ cells. Click here for file

Additional file 2 **This figure shows clonogenic survival assays, analysis of caspase 3 cleavage and quantitative analysis of cell death after knockdown of Survivin.** a: Analysis of clonogenic survival of shSurv-transduced HCT116, U87-MG and SBC-2 cells and isogenic cells with knock out and knockdown of p53, respectively. Note the strong decrease in clonal survival in all cell lines after Survivin knockdown when compared to the shLuc controls. The experiments were repeated twice with similar results. b-c: Western blot analysis of Caspase 3 activation following knockdown of Survivin in (b) U87-MG, U87-MG^shp53^ (72 h) and (c) HCT116, HCT116^p53−/−^ cells at different time points. As control, a total cell lysate from HCT116 cells treated with apoptosis-inducing C2 ceramide (100 μm) is included. d: Quantitative analysis of FACS-assisted DNA-measurements of HCT116, SBC-2 and U87-MG cells and corresponding p53-deficient isogenic cells with knockdown of Survivin or transduction with shLuc control vector. Note the increase in the SubG1-fractions (dead cells) in shSurv-transduced HCT116 and SBC-2 cells when compared to the corresponding shLuc-transduced control cells. (*p < 0.05; **p < 0.01; n = 4). e: Representative images of annexin V – PI stained HCT116 cells 72 h after transduction of shLuc or shSurv, respectively. For apoptosis induction cells were incubated for 24 h with 5 μg/ml puromycin. f: Quantitative analysis of annexin V stained HCT116 and HCT116^p53−/−^ cells transduced with shRNAs targeting Survivin (shSurv) or Luciferase (shLuc) at different time points. Control, HCT116 cells treated for 24 h with 5 μg/ml puromycin; apoptotic cells (annexin V+, PI-); dead cells (annexin V+, PI+; annexin V-, PI+). Data represents mean values and SEM of two independent experiments. Click here for file

Additional file 3 **This movie file shows a merotelic kinetochore spindle assembly in SBC-2 cell with knockdown of Survivin.** This movie shows Z-stacks through a merotelic-attached kinetochore in a SBC-2 cell with knockdown of Survivin. Kinetochores (red) and microtubules (white) were visualized using anti-centromer antibodies (ACA) and a monoclonal anti-Tubulin antibody. Click here for file

Additional file 4 **This figure illustrates numeric and structural chromosomal aberrations following Survivin-RNAi: DAPI-stained metaphase of HCT116**^**p53−/− **^**cells with knockdown of Survivin showing a near hypohexadecaploid (16n) karyotype with dicentric chromosomes (arrows) and ring chromosomes (arrowhead, see magnified regions).** Click here for file

Additional file 5 **This figure depicts site specific accumulation of activated ATM at DNA lesions in U87-MG cells with knockdown of Survivin.** a: Images of U87-MG cells, with knockdown of Survivin and stained for activated ATM S1981 and ɣH2AX. Inlet showing magnification of indicated multinucleated cell with colocalized ɣH2AX and ATM S1981. b: Representative image depicting ɣH2AX and ATM S1981 staining results in shLuc-transduced controls. Colocalization analyses (Coloc) were performed using Fiji’s “Colocalization” algorithm. Magnification bars: 10 μm. Click here for file
