# Supplementary figures and images for: Survivin safeguards chromosome numbers and protects from aneuploidy independently from p53
Source: Mol Cancer. 2014 May 9;13:107. doi: 10.1186/1476-4598-13-107 (PMC4041913; doi:10.1186/1476-4598-13-107)

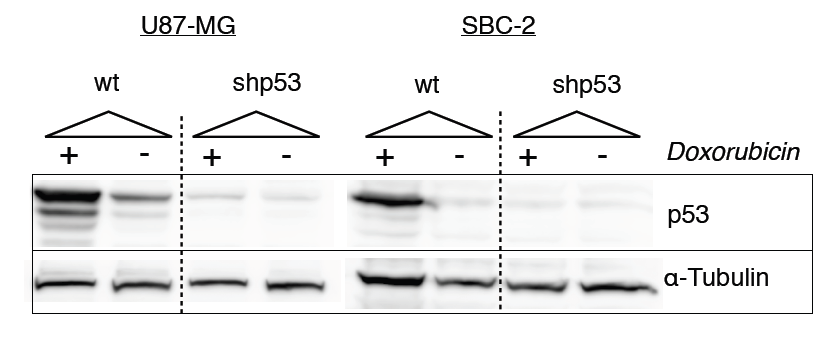

Supplement: Additional file 1 — This figure shows efficiency of p53 knockdown and response to doxorubicin treatment in U87-MG and SBC-2 cells. Western blot analysis showing the knockdown of p53 in glioma and cervix cancer tumor cells. Note that U87-MG and SBC-2 cells accumulate p53 in response to DNA-damaging doxorubicin treatment (0.25 μg/ml) whereas no increase of p53 is observed in U87-MGshp53 and SBC-2shp53 cells. [file 1476-4598-13-107-S1.tiff]

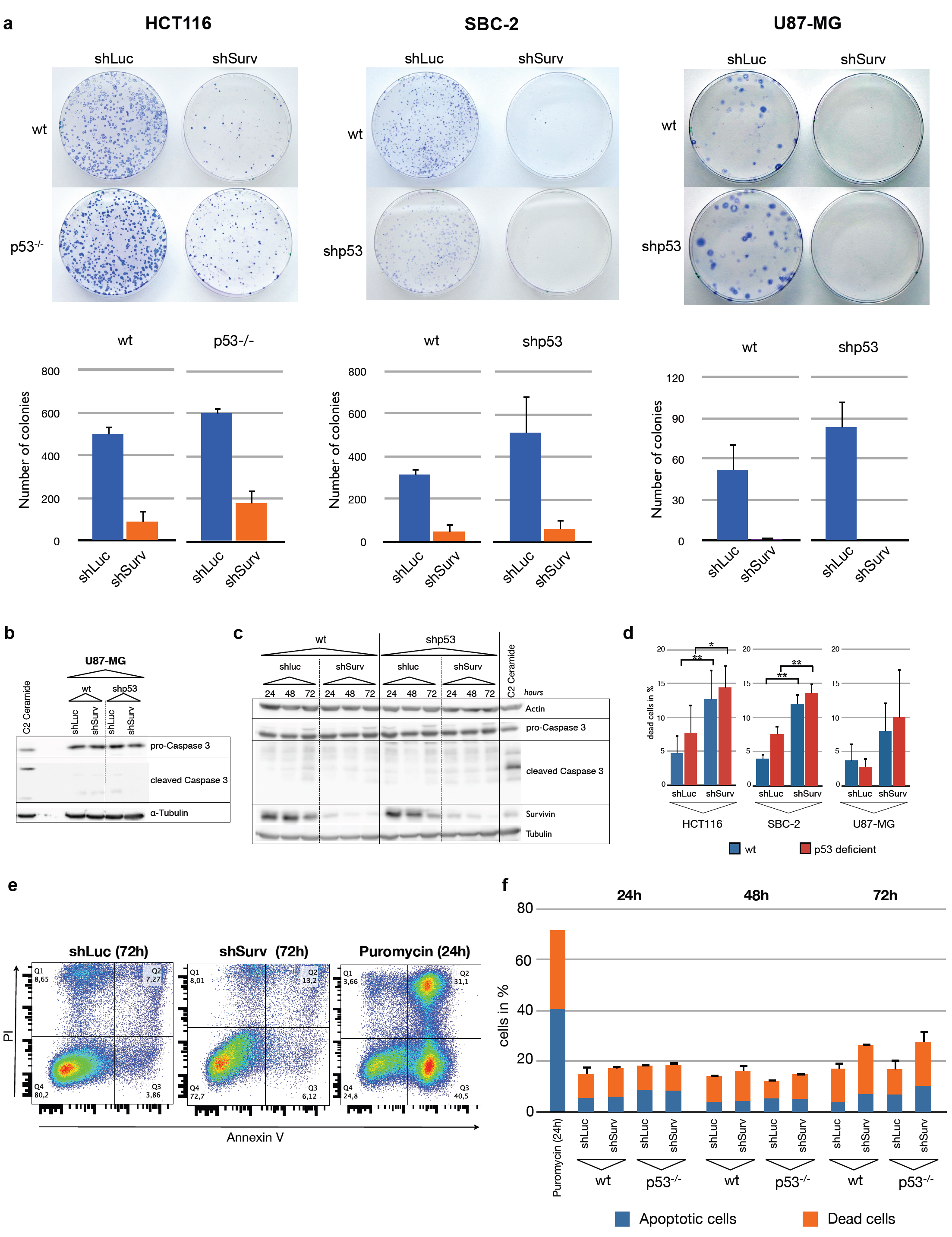

Supplement: Additional file 2 — This figure shows clonogenic survival assays, analysis of caspase 3 cleavage and quantitative analysis of cell death after knockdown of Survivin. a: Analysis of clonogenic survival of shSurv-transduced HCT116, U87-MG and SBC-2 cells and isogenic cells with knock out and knockdown of p53, respectively. Note the strong decrease in clonal survival in all cell lines after Survivin knockdown when compared to the shLuc controls. The experiments were repeated twice with similar results. b-c: Western blot analysis of Caspase 3 activation following knockdown of Survivin in (b) U87-MG, U87-MGshp53 (72 h) and (c) HCT116, HCT116p53−/− cells at different time points. As control, a total cell lysate from HCT116 cells treated with apoptosis-inducing C2 ceramide (100 μm) is included. d: Quantitative analysis of FACS-assisted DNA-measurements of HCT116, SBC-2 and U87-MG cells and corresponding p53-deficient isogenic cells with knockdown of Survivin or transduction with shLuc control vector. Note the increase in the SubG1-fractions (dead cells) in shSurv-transduced HCT116 and SBC-2 cells when compared to the corresponding shLuc-transduced control cells. (*p < 0.05; **p < 0.01; n = 4). e: Representative images of annexin V – PI stained HCT116 cells 72 h after transduction of shLuc or shSurv, respectively. For apoptosis induction cells were incubated for 24 h with 5 μg/ml puromycin. f: Quantitative analysis of annexin V stained HCT116 and HCT116p53−/− cells transduced with shRNAs targeting Survivin (shSurv) or Luciferase (shLuc) at different time points. Control, HCT116 cells treated for 24 h with 5 μg/ml puromycin; apoptotic cells (annexin V+, PI-); dead cells (annexin V+, PI+; annexin V-, PI+). Data represents mean values and SEM of two independent experiments. [file 1476-4598-13-107-S2.tiff]

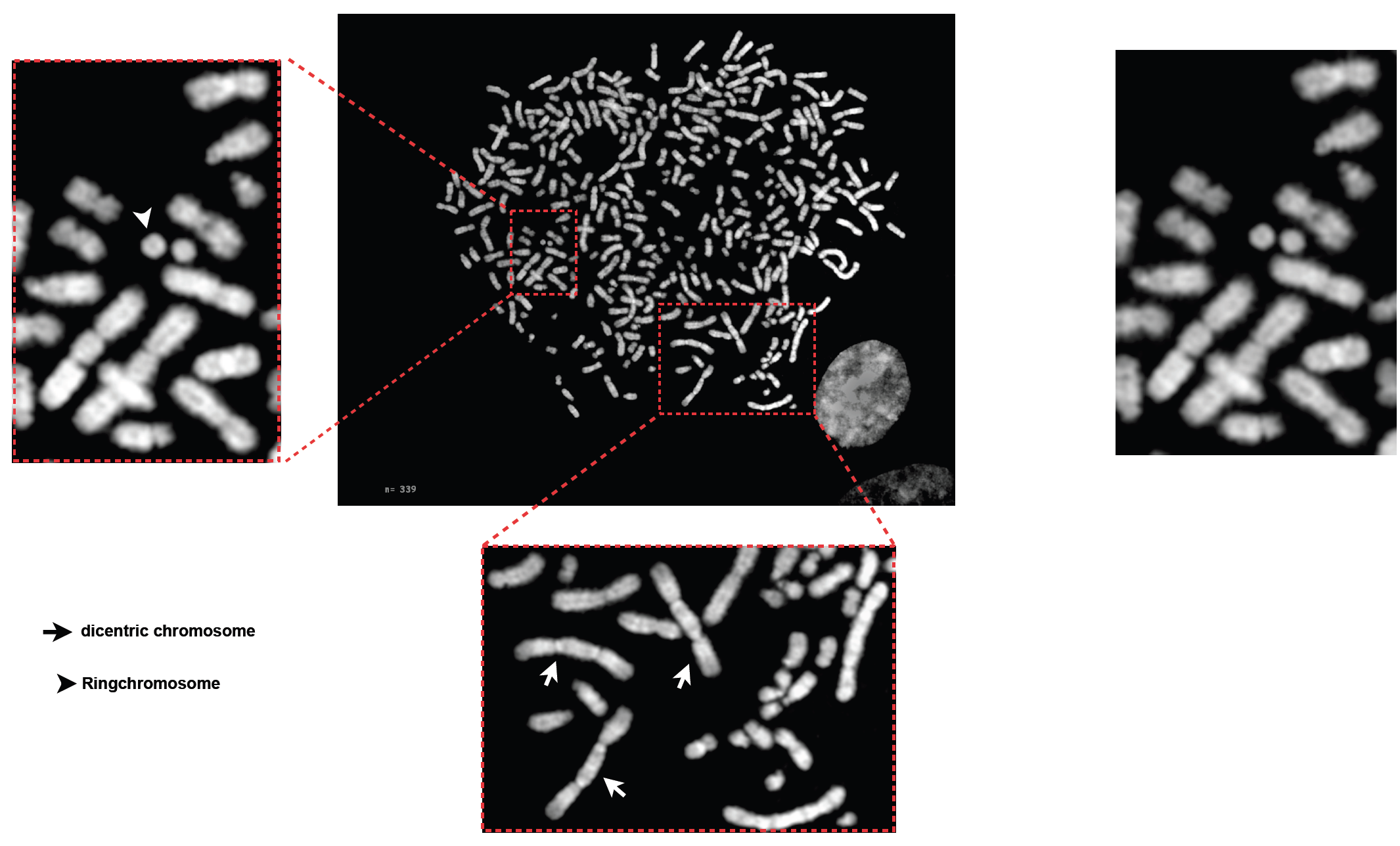

Supplement: Additional file 4 — This figure illustrates numeric and structural chromosomal aberrations following Survivin-RNAi: DAPI-stained metaphase of HCT116 p53−/− cells with knockdown of Survivin showing a near hypohexadecaploid (16n) karyotype with dicentric chromosomes (arrows) and ring chromosomes (arrowhead, see magnified regions). [file 1476-4598-13-107-S4.tiff]

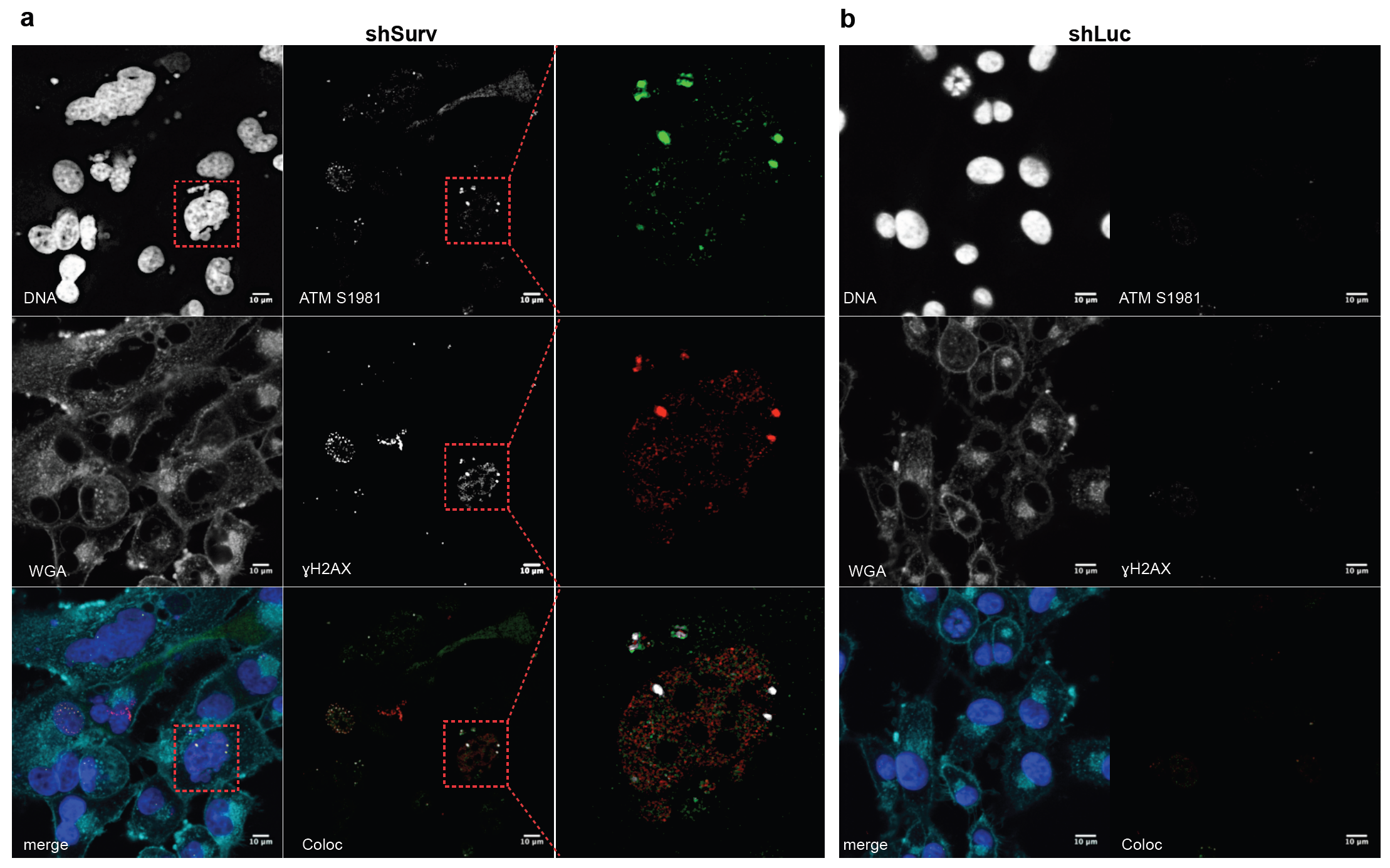

Supplement: Additional file 5 — This figure depicts site specific accumulation of activated ATM at DNA lesions in U87-MG cells with knockdown of Survivin. a: Images of U87-MG cells, with knockdown of Survivin and stained for activated ATM S1981 and ɣH2AX. Inlet showing magnification of indicated multinucleated cell with colocalized ɣH2AX and ATM S1981. b: Representative image depicting ɣH2AX and ATM S1981 staining results in shLuc-transduced controls. Colocalization analyses (Coloc) were performed using Fiji’s “Colocalization” algorithm. Magnification bars: 10 μm. [file 1476-4598-13-107-S5.tiff]
